# Supplementary material for: Diabetic Retinopathy Environment-Wide Association Study (EWAS) in NHANES 2005–2008
Source: J Clin Med. 2020 Nov 12;9(11):3643. doi: 10.3390/jcm9113643 (PMC7696981; doi:10.3390/jcm9113643)
Supplement: Supplementary file 1 [file jcm-09-03643-s001.pdf]

SUPPLEMENTARY TABLES

Table S1. Examination, demographic, and laboratory variables excluded from the study due to having zero variance.

Examination	Demographic	Laboratory
OPDDSNV	FIAPROXY	CARB_E, URDMTOLC
OPDSBCO	RIDSTATR	CARB_E, URDOMOLC
OPDSSNV	SDDSRVYR	CARB_E, URXMTO
OPDUSNV		CARB_E, URXOMO
VIQ110		FOLFMS_E, LBDSF1LC
VIQ130		HEPBD_E, LBDHD
		PAH_E, URDP01LC
		PAH_E, URDP02LC
		PAH_E, URDP04LC
		PAH_E, URDP05LC
		PAH_E, URDP06LC
		PAH_E, URDP17LC
		PERNT_E, URDUP8LC
		PHTHTE_E, URDMEPLC
		PSA_E, KIQ282
		SSHCV_E, SSAHCVI
		UAM_E, URDAAZLC
		UAM_E, URDATZLC
		UAM_E, URDDTZLC
		UAM_E, URDSIMLC
		UAM_E, URDSISLC
		UAM_E, URXAAZ
		UAM_E, URXATZ
		UAM_E, URXDTZ
		UAM_E, URXSIS
		UAM_E, URXSISM
		UHM_E, URDUCSLC
		UPP_E, URDBSMLC
		UPP_E, URDCHSLC
		UPP_E, URDEMMLC
		UPP_E, URDFRMLC
		UPP_E, URDHLSLC
		UPP_E, URDMSMLC
		UPP_E, URDOXSLC
		UPP_E, URDPIMLC
		UPP_E, URDPROLC
		UPP_E, URDRIMLC
		UPP_E, URDSMMLC
		UPP_E, URDSSFLC
		UPP_E, URDTHFLC
		UPP_E, URDTRALC
		UPP_E, URDTRNLC
		UPP_E, URXBSM
		UPP_E, URXCHS
		UPP_E, URXEMM
		UPP_E, URXFRM
		UPP_E, URXHLS
		UPP_E, URXMSM
		UPP_E, URXOXS
		UPP_E, URXPIM
		UPP_E, URXPRO

UPP_E, URXRIM
UPP_E, URXSMM
UPP_E, URXSSF
UPP_E, URXTHF
UPP_E, URXTRA
UPP_E, URXTRN
VID_E, LBDVD3LC
VID_E, LBDVIDLC
VOCWB_E, LBD4CELC
VOCWB_E, LBDV1ALC
VOCWB_E, LBDV1ELC
VOCWB_E, LBDV2CLC
VOCWB_E, LBDV2ELC
VOCWB_E, LBDV2PLC
VOCWB_E, LBDV2TLC
VOCWB_E, LBDV3BLC
VOCWB_E, LBDV4ELC
VOCWB_E, LBDVCBLC
VOCWB_E, LBDVDELC
VOCWB_E, LBDVHELC
VOCWB_E, LBDVTPLC
VOCWB_E, LBDVTPLC
VOCWB_E, LBX4CE
VOCWB_E, LBXV1A
VOCWB_E, LBXV1E
VOCWB_E, LBXV2C
VOCWB_E, LBXV2E
VOCWB_E, LBXV2P
VOCWB_E, LBXV2T
VOCWB_E, LBXV3B
VOCWB_E, LBXV4E
VOCWB_E, LBXVCB
VOCWB_E, LBXVDE
VOCWB_E, LBXVHE
VOCWB_E, LBXVTP

Table S2. Statistically significant principal components (PCs) associated with diabetic retinopathy (DR).

PC	Beta	Standard Error	Z	*p*-Value	OR (95% CI)
PC1	−0.23	0.03	−6.73	<0·0001	0.79 (95% CI: 0.74–0.85)
PC9	0.19	0.05	3.92	<0·0001	1.21 (95% CI: 1.1–1.33)
PC211	−16.23	4.53	−3.58	0.0003	0 (95% CI: 0–0)
PC3	−0.14	0.04	−3.54	0.0004	0.87 (95% CI: 0.81–0.94)
PC2	−0.10	0.03	−3.20	0.001	0.9 (95% CI: 0.85–0.96)
PC21	0.20	0.07	3.02	0.003	1.22 (95% CI: 1.07–1.39)
PC27	0.21	0.08	2.80	0.005	1.24 (95% CI: 1.07–1.43)
PC110	0.48	0.17	2.76	0.006	1.62 (95% CI: 1.15–2.28)
PC8	−0.13	0.05	−2.66	0.008	0.88 (95% CI: 0.8–0.97)
PC142	−0.65	0.24	−2.65	0.008	0.52 (95% CI: 0.32–0.84)
PC38	0.22	0.09	2.49	0.01	1.24 (95% CI: 1.05–1.47)
PC34	0.19	0.08	2.26	0.02	1.21 (95% CI: 1.03–1.43)
PC25	0.16	0.07	2.24	0.03	1.18 (95% CI: 1.02–1.36)
PC105	−0.35	0.16	−2.16	0.03	0.7 (95% CI: 0.51–0.97)
PC117	−0.38	0.18	−2.12	0.03	0.68 (95% CI: 0.48–0.97)
PC30	0.17	0.08	2.10	0.04	1.18 (95% CI: 1.01–1.38)
PC152	−0.56	0.27	−2.08	0.04	0.57 (95% CI: 0.34–0.97)
PC37	−0.17	0.09	−2.04	0.04	0.84 (95% CI: 0.71–0.99)
PC13	0.10	0.05	1.88	0.06	1.11 (95% CI: 1–1.23)

PC19	−0.12	0.06	−1.88	0.06	0.89 (95% CI: 0.79–1.01)
PC10	0.09	0.05	1.85	0.06	1.1 (95% CI: 0.99–1.21)
PC90	−0.26	0.14	−1.82	0.07	0.77 (95% CI: 0.58–1.02)
PC148	−0.46	0.25	−1.80	0.07	0.63 (95% CI: 0.38–1.04)
PC29	−0.14	0.08	−1.80	0.07	0.87 (95% CI: 0.75–1.01)
PC185	0.89	0.50	1.78	0.07	2.44 (95% CI: 0.91–6.52)
PC70	−0.21	0.12	−1.76	0.08	0.81 (95% CI: 0.64–1.02)
PC17	−0.11	0.06	−1.76	0.08	0.9 (95% CI: 0.8–1.01)
PC35	0.15	0.09	1.72	0.09	1.16 (95% CI: 0.98–1.39)
PC208	−4.82	2.84	−1.70	0.09	0.01 (95% CI: 0–2.11)
PC205	−2.43	1.45	−1.68	0.09	0.09 (95% CI: 0.01–1.5)
PC62	−0.18	0.11	−1.66	0.10	0.83 (95% CI: 0.67–1.03)
PC114	−0.29	0.18	−1.65	0.10	0.75 (95% CI: 0.53–1.06)
PC98	−0.25	0.15	−1.64	0.10	0.78 (95% CI: 0.58–1.05)
PC46	0.15	0.09	1.63	0.10	1.17 (95% CI: 0.97–1.4)
PC94	−0.23	0.15	−1.55	0.12	0.8 (95% CI: 0.6–1.06)
PC18	−0.09	0.06	−1.54	0.12	0.91 (95% CI: 0.81–1.03)
PC60	−0.17	0.11	−1.52	0.13	0.85 (95% CI: 0.68–1.05)
PC45	−0.14	0.09	−1.50	0.13	0.87 (95% CI: 0.72–1.04)
PC197	1.00	0.68	1.47	0.14	2.72 (95% CI: 0.72–10.29)
PC118	0.26	0.18	1.42	0.15	1.3 (95% CI: 0.91–1.85)
PC77	0.18	0.13	1.41	0.16	1.2 (95% CI: 0.93–1.54)
PC150	−0.36	0.26	−1.40	0.16	0.7 (95% CI: 0.42–1.16)
PC89	−0.19	0.14	−1.36	0.17	0.83 (95% CI: 0.63–1.09)
PC184	0.68	0.52	1.32	0.19	1.98 (95% CI: 0.72–5.46)
PC79	−0.17	0.13	−1.32	0.19	0.84 (95% CI: 0.65–1.09)
PC20	−0.09	0.07	−1.31	0.19	0.92 (95% CI: 0.81–1.04)
PC64	−0.15	0.11	−1.31	0.19	0.86 (95% CI: 0.69–1.08)
PC74	−0.16	0.12	−1.30	0.19	0.85 (95% CI: 0.68–1.08)
PC216	120.42	92.41	1.30	0.19	1.98482520108455E+52 (95% CI: 0–9.137746010503E+130)
PC93	0.20	0.15	1.30	0.19	1.22 (95% CI: 0.9–1.64)
PC47	−0.13	0.10	−1.30	0.19	0.88 (95% CI: 0.72–1.07)
PC86	−0.18	0.14	−1.29	0.20	0.84 (95% CI: 0.64–1.1)
PC158	0.36	0.29	1.26	0.21	1.44 (95% CI: 0.82–2.52)
PC124	−0.24	0.19	−1.24	0.21	0.79 (95% CI: 0.54–1.15)
PC33	0.10	0.08	1.24	0.21	1.11 (95% CI: 0.94–1.3)
PC11	0.06	0.05	1.24	0.22	1.06 (95% CI: 0.96–1.18)
PC200	1.04	0.85	1.23	0.22	2.84 (95% CI: 0.54–14.98)
PC16	−0.07	0.06	−1.22	0.22	0.93 (95% CI: 0.83–1.04)
PC190	−0.68	0.56	−1.22	0.22	0.51 (95% CI: 0.17–1.52)
PC138	−0.28	0.23	−1.21	0.23	0.76 (95% CI: 0.49–1.19)
PC68	0.14	0.12	1.18	0.24	1.15 (95% CI: 0.91–1.44)
PC175	0.42	0.36	1.15	0.25	1.52 (95% CI: 0.75–3.07)
PC66	0.13	0.11	1.15	0.25	1.14 (95% CI: 0.91–1.43)
PC39	−0.10	0.09	−1.13	0.26	0.9 (95% CI: 0.75–1.08)
PC202	1.06	0.94	1.13	0.26	2.87 (95% CI: 0.46–18.04)
PC87	−0.16	0.14	−1.12	0.26	0.86 (95% CI: 0.65–1.12)
PC157	0.31	0.28	1.12	0.26	1.36 (95% CI: 0.79–2.36)
PC134	0.24	0.22	1.11	0.27	1.28 (95% CI: 0.83–1.96)
PC122	0.21	0.19	1.09	0.27	1.23 (95% CI: 0.85–1.78)
PC192	−0.63	0.57	−1.09	0.27	0.53 (95% CI: 0.17–1.65)
PC176	0.39	0.37	1.06	0.29	1.48 (95% CI: 0.72–3.04)
PC144	−0.25	0.24	−1.05	0.29	0.78 (95% CI: 0.48–1.25)
PC104	−0.17	0.16	−1.04	0.30	0.84 (95% CI: 0.61–1.16)
PC214	−17.04	16.48	−1.03	0.30	0 (95% CI: 0–4213127.42)
PC57	0.11	0.11	1.03	0.30	1.11 (95% CI: 0.91–1.37)
PC149	−0.27	0.26	−1.02	0.31	0.77 (95% CI: 0.46–1.28)
PC102	0.15	0.15	1.00	0.32	1.16 (95% CI: 0.87–1.57)

PC153	−0.26	0.26	−0.99	0.32	0.77 (95% CI: 0.46–1.29)
PC12	0.05	0.05	0.98	0.33	1.05 (95% CI: 0.95–1.17)
PC212	5.01	5.28	0.95	0.34	149.65 (95% CI: 0–4697021.47)
PC28	−0.07	0.08	−0.95	0.34	0.93 (95% CI: 0.8–1.08)
PC26	−0.07	0.07	−0.93	0.35	0.93 (95% CI: 0.81–1.08)
PC130	−0.19	0.21	−0.93	0.35	0.83 (95% CI: 0.55–1.24)
PC54	−0.09	0.10	−0.91	0.36	0.91 (95% CI: 0.74–1.11)
PC42	−0.08	0.09	−0.90	0.37	0.92 (95% CI: 0.77–1.1)
PC187	−0.47	0.53	−0.88	0.38	0.63 (95% CI: 0.22–1.77)
PC126	−0.17	0.19	−0.88	0.38	0.84 (95% CI: 0.58–1.23)
PC168	−0.28	0.32	−0.88	0.38	0.76 (95% CI: 0.41–1.41)
PC188	0.48	0.55	0.87	0.38	1.61 (95% CI: 0.55–4.71)
PC145	0.21	0.24	0.87	0.38	1.23 (95% CI: 0.77–1.98)
PC215	10.14	11.66	0.87	0.38	25387.49 (95% CI: 0–212202584172954)
PC196	0.57	0.66	0.86	0.39	1.76 (95% CI: 0.49–6.37)
PC113	−0.15	0.18	−0.86	0.39	0.86 (95% CI: 0.61–1.21)
PC95	0.13	0.15	0.86	0.39	1.14 (95% CI: 0.85–1.52)
PC217	########	########	0.85	0.39	NA
PC108	0.14	0.17	0.85	0.40	1.15 (95% CI: 0.83–1.59)
PC112	−0.14	0.17	−0.83	0.41	0.87 (95% CI: 0.62–1.21)
PC65	−0.09	0.11	−0.83	0.41	0.91 (95% CI: 0.73–1.14)
PC195	0.53	0.64	0.83	0.41	1.71 (95% CI: 0.48–6.03)
PC167	−0.26	0.32	−0.83	0.41	0.77 (95% CI: 0.41–1.43)
PC88	0.12	0.14	0.83	0.41	1.12 (95% CI: 0.85–1.48)
PC67	0.10	0.12	0.82	0.41	1.1 (95% CI: 0.88–1.38)
PC171	−0.27	0.34	−0.79	0.43	0.76 (95% CI: 0.39–1.49)
PC136	−0.18	0.22	−0.79	0.43	0.84 (95% CI: 0.54–1.3)
PC15	−0.04	0.06	−0.77	0.44	0.96 (95% CI: 0.85–1.07)
PC191	0.43	0.57	0.76	0.45	1.54 (95% CI: 0.5–4.73)
PC166	0.24	0.31	0.75	0.45	1.27 (95% CI: 0.68–2.34)
PC209	2.14	2.84	0.75	0.45	8.48 (95% CI: 0.03–2222.54)
PC225	########	########	−0.72	0.47	NA
PC99	−0.11	0.15	−0.72	0.47	0.9 (95% CI: 0.66–1.21)
PC23	−0.05	0.07	−0.71	0.47	0.95 (95% CI: 0.83–1.09)
PC143	0.17	0.24	0.71	0.48	1.19 (95% CI: 0.74–1.89)
PC40	−0.06	0.09	−0.70	0.48	0.94 (95% CI: 0.79–1.12)
PC170	−0.23	0.33	−0.70	0.48	0.79 (95% CI: 0.42–1.51)
PC181	0.29	0.42	0.68	0.50	1.33 (95% CI: 0.59–3.02)
PC219	########	########	−0.68	0.50	NA
PC44	−0.06	0.09	−0.66	0.51	0.94 (95% CI: 0.78–1.13)
PC120	0.12	0.19	0.66	0.51	1.13 (95% CI: 0.78–1.63)
PC75	−0.08	0.12	−0.65	0.51	0.92 (95% CI: 0.72–1.18)
PC147	−0.16	0.25	−0.64	0.52	0.85 (95% CI: 0.52–1.39)
PC220	########	########	0.63	0.53	NA
PC51	0.06	0.10	0.63	0.53	1.06 (95% CI: 0.88–1.29)
PC84	0.08	0.14	0.63	0.53	1.09 (95% CI: 0.83–1.42)
PC164	−0.18	0.30	−0.60	0.55	0.83 (95% CI: 0.46–1.51)
PC137	0.14	0.22	0.60	0.55	1.14 (95% CI: 0.74–1.77)
PC32	−0.05	0.08	−0.60	0.55	0.95 (95% CI: 0.81–1.12)
PC180	0.24	0.40	0.59	0.55	1.27 (95% CI: 0.58–2.8)
PC125	0.11	0.19	0.57	0.57	1.12 (95% CI: 0.76–1.63)
PC146	−0.14	0.25	−0.57	0.57	0.87 (95% CI: 0.53–1.41)
PC154	0.15	0.27	0.56	0.58	1.16 (95% CI: 0.69–1.97)
PC119	−0.10	0.19	−0.54	0.59	0.9 (95% CI: 0.63–1.3)
PC73	0.07	0.12	0.54	0.59	1.07 (95% CI: 0.84–1.35)
PC201	−0.46	0.87	−0.53	0.59	0.63 (95% CI: 0.11–3.47)
PC36	0.04	0.08	0.53	0.60	1.05 (95% CI: 0.89–1.23)
PC123	0.10	0.19	0.53	0.60	1.11 (95% CI: 0.76–1.61)
PC100	−0.08	0.15	−0.52	0.60	0.92 (95% CI: 0.68–1.25)

PC174	−0.18	0.35	−0.51	0.61	0.84 (95% CI: 0.42–1.67)
PC50	−0.05	0.10	−0.50	0.62	0.95 (95% CI: 0.78–1.16)
PC151	0.13	0.26	0.49	0.62	1.14 (95% CI: 0.68–1.9)
PC226	########	########	0.49	0.63	NA
PC199	0.36	0.77	0.47	0.64	1.44 (95% CI: 0.32–6.51)
PC182	−0.20	0.43	−0.46	0.64	0.82 (95% CI: 0.36–1.89)
PC69	0.05	0.12	0.46	0.65	1.06 (95% CI: 0.84–1.33)
PC169	−0.15	0.32	−0.45	0.65	0.86 (95% CI: 0.46–1.63)
PC179	0.18	0.40	0.45	0.65	1.2 (95% CI: 0.55–2.6)
PC97	−0.07	0.15	−0.44	0.66	0.94 (95% CI: 0.7–1.26)
PC173	−0.15	0.35	−0.43	0.67	0.86 (95% CI: 0.44–1.7)
PC189	0.23	0.55	0.42	0.67	1.26 (95% CI: 0.43–3.73)
PC133	−0.09	0.21	−0.42	0.68	0.92 (95% CI: 0.61–1.39)
PC71	0.05	0.12	0.41	0.68	1.05 (95% CI: 0.83–1.33)
PC224	########	########	0.41	0.68	NA
PC160	0.12	0.29	0.41	0.68	1.13 (95% CI: 0.63–2.01)
PC78	−0.05	0.13	−0.40	0.69	0.95 (95% CI: 0.74–1.22)
PC76	0.05	0.13	0.40	0.69	1.05 (95% CI: 0.82–1.34)
PC156	−0.11	0.28	−0.40	0.69	0.9 (95% CI: 0.52–1.54)
PC163	−0.12	0.30	−0.39	0.69	0.89 (95% CI: 0.49–1.61)
PC109	0.07	0.17	0.39	0.69	1.07 (95% CI: 0.77–1.49)
PC162	0.12	0.30	0.39	0.70	1.12 (95% CI: 0.63–2.01)
PC59	0.04	0.11	0.39	0.70	1.04 (95% CI: 0.84–1.29)
PC58	−0.04	0.10	−0.38	0.70	0.96 (95% CI: 0.78–1.18)
PC193	0.24	0.63	0.38	0.70	1.27 (95% CI: 0.37–4.4)
PC115	−0.07	0.18	−0.37	0.71	0.94 (95% CI: 0.66–1.32)
PC198	−0.26	0.71	−0.37	0.71	0.77 (95% CI: 0.19–3.09)
PC177	−0.14	0.38	−0.36	0.72	0.87 (95% CI: 0.42–1.82)
PC72	−0.04	0.12	−0.36	0.72	0.96 (95% CI: 0.75–1.21)
PC91	−0.05	0.14	−0.36	0.72	0.95 (95% CI: 0.72–1.26)
PC22	0.02	0.07	0.35	0.73	1.02 (95% CI: 0.9–1.17)
PC41	0.03	0.09	0.35	0.73	1.03 (95% CI: 0.86–1.23)
PC101	0.05	0.16	0.35	0.73	1.06 (95% CI: 0.78–1.43)
PC165	−0.11	0.31	−0.34	0.73	0.9 (95% CI: 0.49–1.65)
PC139	−0.08	0.23	−0.33	0.74	0.93 (95% CI: 0.59–1.45)
PC210	−1.07	3.21	−0.33	0.74	0.34 (95% CI: 0–187.7)
PC128	−0.07	0.20	−0.33	0.74	0.94 (95% CI: 0.63–1.38)
PC129	0.07	0.20	0.32	0.75	1.07 (95% CI: 0.72–1.59)
PC83	−0.04	0.13	−0.31	0.76	0.96 (95% CI: 0.74–1.25)
PC132	−0.06	0.21	−0.30	0.76	0.94 (95% CI: 0.62–1.42)
PC5	−0.01	0.04	−0.29	0.77	0.99 (95% CI: 0.92–1.07)
PC4	−0.01	0.04	−0.28	0.78	0.99 (95% CI: 0.92–1.06)
PC43	−0.03	0.09	−0.28	0.78	0.97 (95% CI: 0.81–1.17)
PC159	0.08	0.29	0.27	0.79	1.08 (95% CI: 0.61–1.9)
PC82	−0.03	0.13	−0.27	0.79	0.97 (95% CI: 0.75–1.25)
PC6	−0.01	0.04	−0.26	0.79	0.99 (95% CI: 0.91–1.08)
PC31	−0.02	0.08	−0.26	0.80	0.98 (95% CI: 0.84–1.14)
PC49	−0.03	0.10	−0.26	0.80	0.98 (95% CI: 0.81–1.18)
PC127	0.05	0.20	0.26	0.80	1.05 (95% CI: 0.71–1.55)
PC161	0.07	0.29	0.25	0.80	1.08 (95% CI: 0.61–1.91)
PC85	−0.03	0.14	−0.24	0.81	0.97 (95% CI: 0.74–1.26)
PC111	0.04	0.17	0.23	0.82	1.04 (95% CI: 0.74–1.45)
PC203	0.22	1.02	0.22	0.83	1.25 (95% CI: 0.17–9.23)
PC55	0.02	0.10	0.21	0.83	1.02 (95% CI: 0.83–1.26)
PC207	0.30	1.45	0.20	0.84	1.34 (95% CI: 0.08–23.25)
PC141	−0.05	0.23	−0.20	0.84	0.95 (95% CI: 0.6–1.51)
PC223	########	########	−0.18	0.85	NA
PC14	−0.01	0.06	−0.18	0.86	0.99 (95% CI: 0.89–1.1)
PC7	−0.01	0.04	−0.18	0.86	0.99 (95% CI: 0.91–1.08)

PC56	0.02	0.10	0.17	0.86	1.02 (95% CI: 0.83–1.25)
PC121	−0.03	0.19	−0.17	0.86	0.97 (95% CI: 0.67–1.4)
PC221	########	########	−0.17	0.87	NA
PC186	0.08	0.51	0.17	0.87	1.09 (95% CI: 0.4–2.93)
PC80	0.02	0.13	0.15	0.88	1.02 (95% CI: 0.79–1.32)
PC131	−0.03	0.21	−0.15	0.88	0.97 (95% CI: 0.64–1.46)
PC222	########	########	−0.15	0.88	NA
PC52	−0.01	0.10	−0.13	0.89	0.99 (95% CI: 0.81–1.2)
PC140	0.03	0.23	0.13	0.90	1.03 (95% CI: 0.65–1.63)
PC48	0.01	0.10	0.13	0.90	1.01 (95% CI: 0.84–1.22)
PC155	−0.03	0.27	−0.12	0.90	0.97 (95% CI: 0.56–1.65)
PC172	−0.04	0.34	−0.12	0.90	0.96 (95% CI: 0.49–1.88)
PC106	0.02	0.16	0.12	0.91	1.02 (95% CI: 0.74–1.4)
PC116	−0.02	0.18	−0.11	0.91	0.98 (95% CI: 0.69–1.39)
PC63	−0.01	0.11	−0.10	0.92	0.99 (95% CI: 0.79–1.23)
PC81	−0.01	0.13	−0.09	0.93	0.99 (95% CI: 0.76–1.28)
PC194	0.05	0.61	0.08	0.94	1.05 (95% CI: 0.32–3.48)
PC135	−0.01	0.22	−0.07	0.95	0.99 (95% CI: 0.64–1.52)
PC92	−0.01	0.15	−0.06	0.95	0.99 (95% CI: 0.75–1.32)
PC183	−0.02	0.44	−0.05	0.96	0.98 (95% CI: 0.41–2.31)
PC107	0.01	0.17	0.05	0.96	1.01 (95% CI: 0.73–1.39)
PC61	0.00	0.11	0.04	0.97	1 (95% CI: 0.81–1.24)
PC53	0.00	0.10	−0.03	0.98	1 (95% CI: 0.82–1.21)
PC213	0.19	6.20	0.03	0.98	1.21 (95% CI: 0–228449.56)
PC178	−0.01	0.38	−0.03	0.98	0.99 (95% CI: 0.47–2.1)
PC96	0.00	0.15	0.03	0.98	1 (95% CI: 0.75–1.35)
PC206	−0.03	1.31	−0.02	0.98	0.97 (95% CI: 0.07–12.6)
PC103	0.00	0.16	0.02	0.99	1 (95% CI: 0.74–1.37)
PC218	########	########	−0.01	0.99	NA
PC24	0.00	0.07	0.00	1.00	1 (95% CI: 0.87–1.14)
PC204	0.00	1.23	0.00	1.00	1 (95% CI: 0.09–11.13)

Notes: Principal component analysis was performed on laboratory variables in an unsupervised fashion, with resulting PCs regressed against DR outcome.

Table S3. RandomForest™-selected features for replication cohort NHANES 2005-2006.

Marker	Mean decrease accuracy	Mean decrease gini
Glycohemoglobin (%)	16.96	18.06
Pyridoxal 5'-phosphate (nmol/L)	8.15	5.24
Total Cholesterol (mmol/L)	8.14	6.33
Cholesterol (mmol/L)	7.53	6.10
Blood urea nitrogen (mmol/L)	5.62	5.14
Diastolic blood pressure	4.76	5.94
Albumin, urine (mg/L)	4.25	6.00
Hematocrit (%)	4.18	3.35
Lactate dehydrogenase LDH (U/L)	3.97	4.44
Mono-(2-ethyl-5-hydroxyhexyl) phthalate	3.72	1.75
Uranium, urinary (µg/L)	3.57	2.55
2-amnothiazolne-4-carbxylic acid(ng/mL)	3.51	3.25
Mono-n-methyl phthalate	3.22	1.85
Urinary nitrate (ng/mL)	3.20	7.25
Blood Carbon Tetrachloride (ng/mL)	3.06	0.70
Perchlorate, water (ng/mL)	2.61	3.50
Iron, refigerated (µmol/L)	2.59	4.87
Potassium (mmol/L)	2.47	4.06
Nitrate, water (ng/mL)	2.35	2.61
Red blood cell count (million cells/µL)	1.89	4.61
Mono-2-ethyl-5-carboxypentyl phthalate	1.67	2.02
Hemoglobin (g/dL)	1.47	2.47

2-hydroxyphenanthrene (ng/L)	1.24	2.50
Dimethyldithiophosphate (µg/L)	1.05	1.24
Alkaline phosphotase (U/L)	0.80	4.56
Propyl paraben (ng/ml)	0.80	3.02

Notes: The model was initially trained on all laboratory variables in an unsupervised fashion, with Kappa-based model tuning to select the optimum values for "mtry" (the ideal number of variables to randomly sample) and "ntrees" (the ideal number of trees). Only features contributing >1% mean decrease in accuracy from initial model were retained.

Supplementary figures

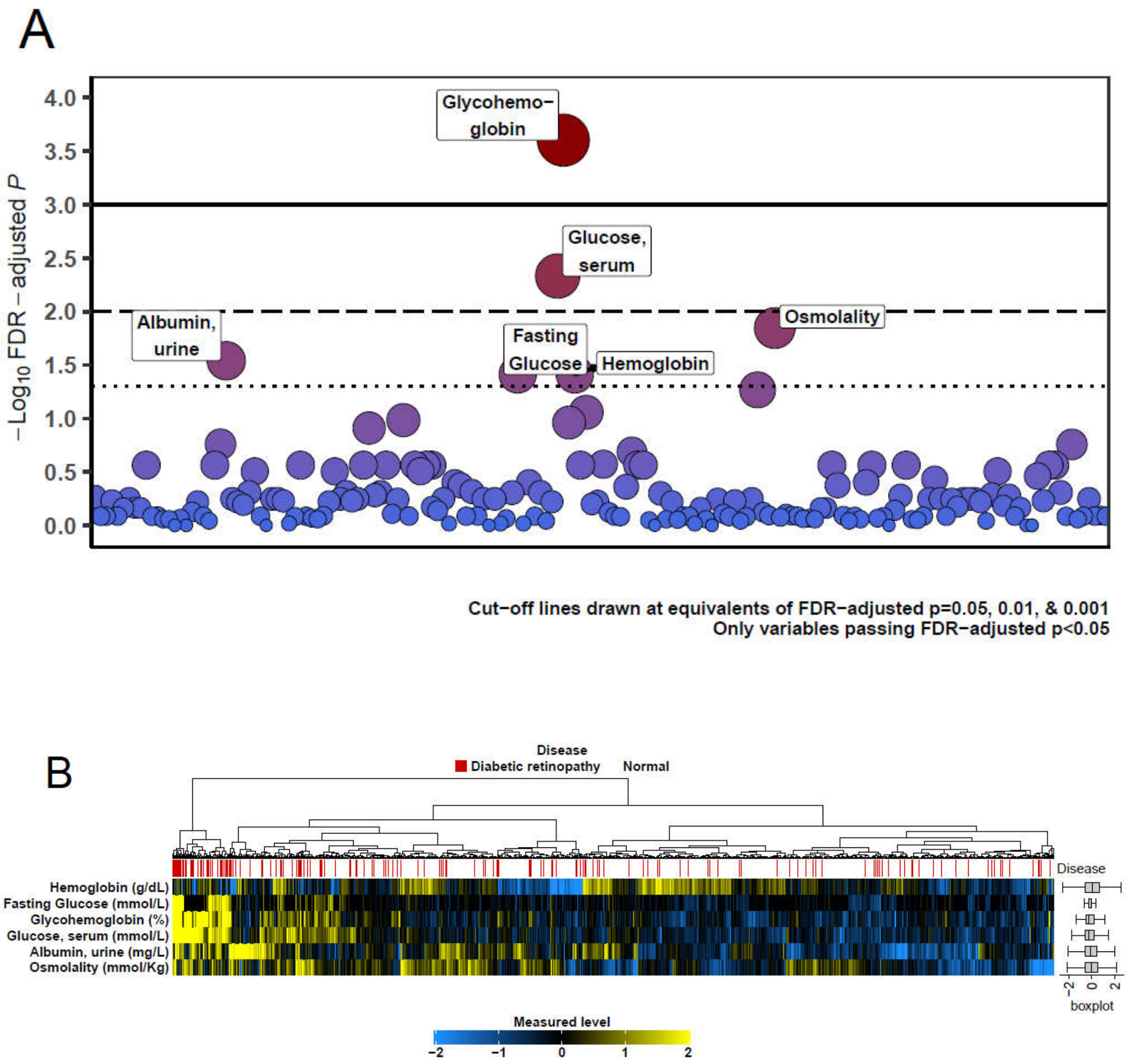

Figure S1. Laboratory variables statistically significantly associated with diabetic retinopathy.

Variables were tested in a univariate fashion, with all *p*-values then adjusted for false discovery rate (FDR). Six variables reached statistical significance at FDR-adjusted $p \leq 0{\cdot}05$. **A)** Manhattan plot of laboratory variables associated with diabetic retinopathy from univariate testing. Variables were tested via a compute-parallelized procedure, with *p*-values then adjusted for false discovery rate (FDR). Six variables reached statistical significance at FDR-adjusted $p \leq 0.05$. Y-axis is plotting $-\log_{10}$ (FDR-adjusted *p*-value). **B)** clustering and heatmap.

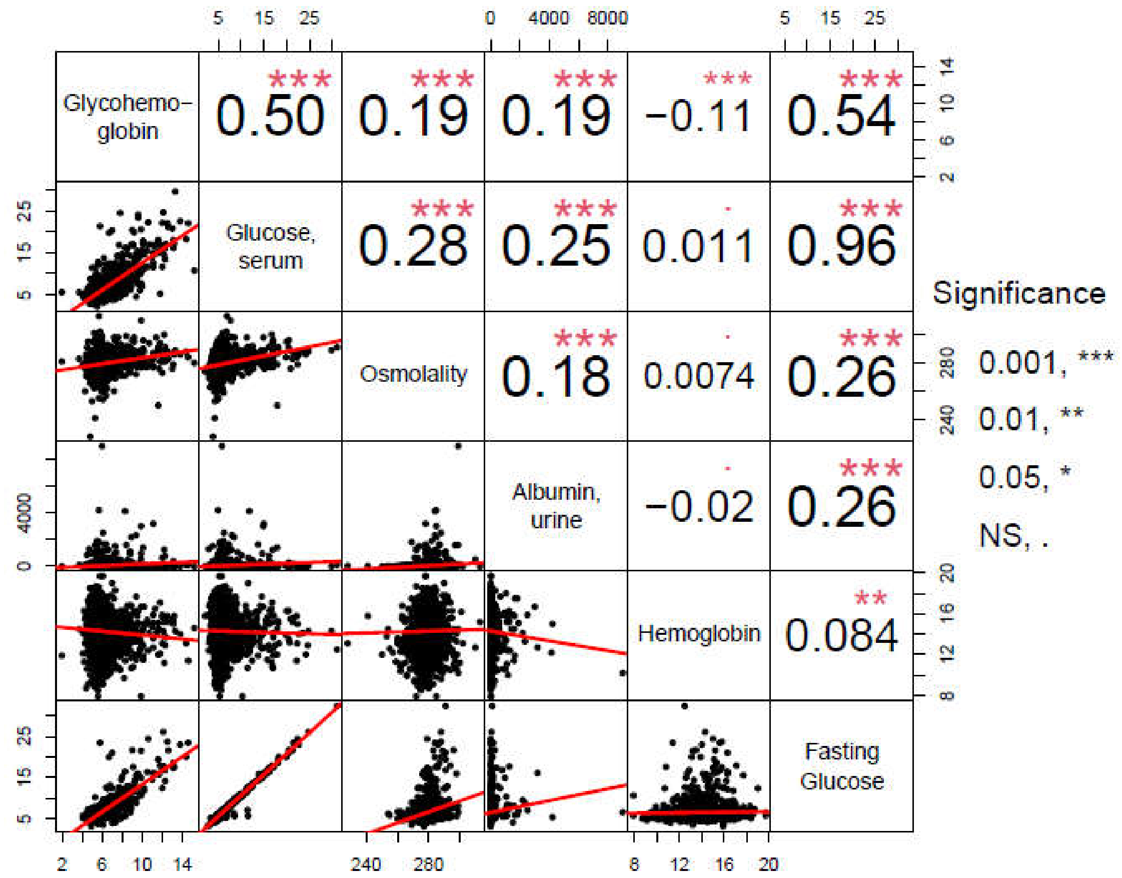

Figure S2. Inter-correlation among laboratory variables statistically significantly associated with diabetic retinopathy.

Bottom triangle, scatter plots with linear regression fit line. Top triangle, Spearman ϱ (rho) coefficients, with *p*-value from correlation test indicated by asterisks.

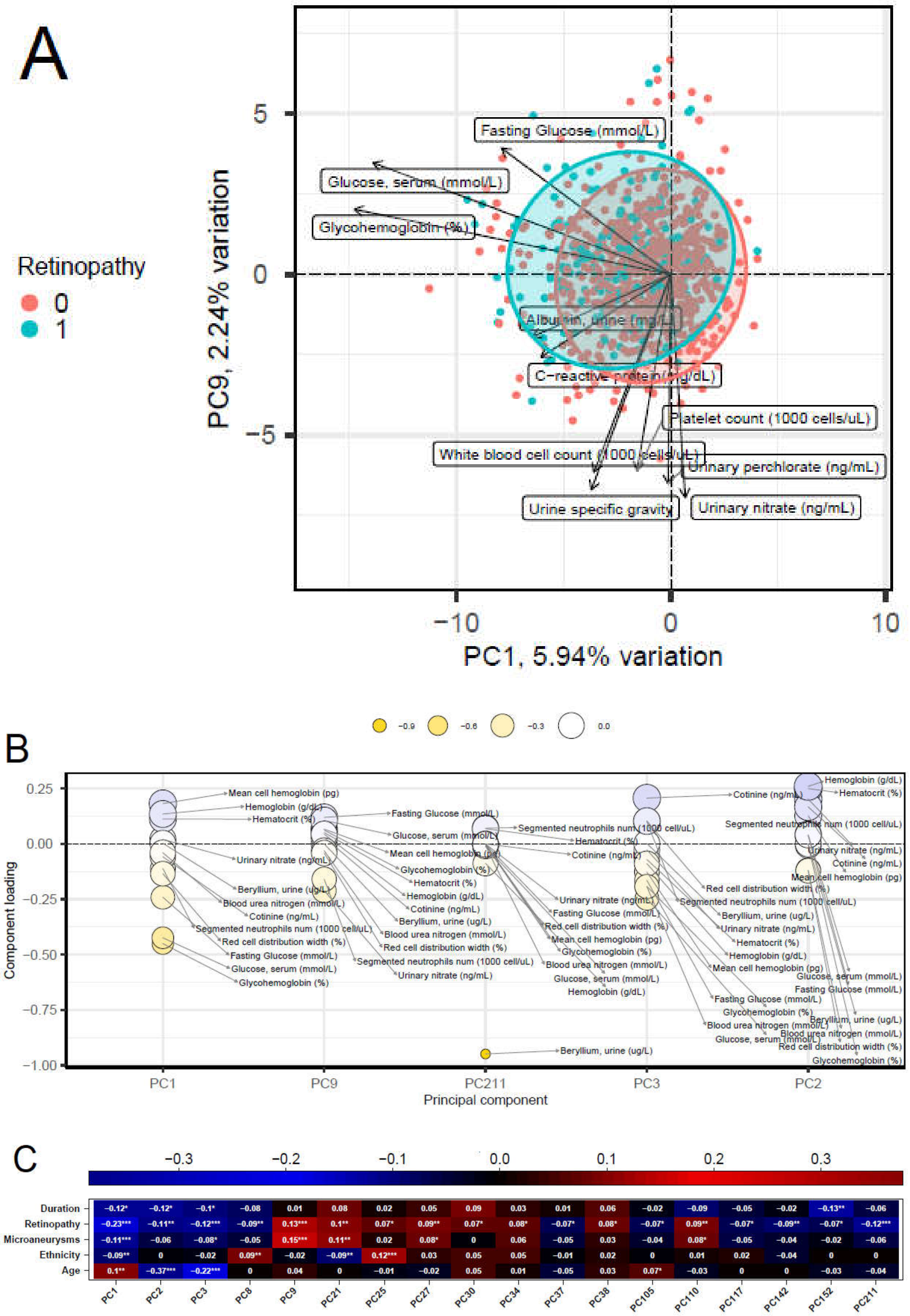

Figure S3. Principal component analysis (PCA) highlights key sources of variation in the cohort.

PCA was performed in an unsupervised fashion on laboratory variables. A, bi-plot of PC1 versus PC9, the top two PCs statistically significantly associated with diabetic retinopathy (DR) (see **Supplementary Table 2**), reveals HbA1c, CRP, and others as key drivers of variation along these PCs. 0, no retinopathy; 1, retinopathy. B, top variables contributing to variation along top five PCs statistically significantly associated with DR. C, "eigencorplot" comparing covariates (age, ethnicity, and diabetes duration), retinopathy (as DR), and the top retinal lesion (microaneurysms) against all PCs statistically significantly associated with DR. The statistically significant correlations highlight relevant usage of these covariates during univariate testing. Pearson correlation values shown, with significancies: ***, $p \leq 0{\cdot}001$; **, $p \leq 0{\cdot}01$; *, $p \leq 0{\cdot}05$.
